# Prediction of personalised postprandial glycaemic response in type 1 diabetes mellitus

**DOI:** 10.3389/fendo.2024.1423303

**Published:** 2024-07-09

**Authors:** Xin Xiong, Yuxin Xue, Yunying Cai, Jianfeng He, Heng Su

**Affiliations:** ^1^ Faculty of Information Engineering and Automation, Kunming University of Science and Technology, Kunming, China; ^2^ Department of Endocrinology, The First People’s Hospital of Yunnan Province, The Affiliated Hospital of Kunming University of Science and Technology, Kunming, China

**Keywords:** type 1 diabetes, postprandial glycaemic response, personalized nutrition, continuous glucose monitors, dietary nutrients

## Abstract

**Objectives:**

Patients with type 1 diabetes (T1D) face unique challenges in glycaemic control due to the complexity and uniqueness of the dietary structure in China, especially in terms of postprandial glycaemic response (PPGR). This study aimed to establish a personalized model for predicting PPGR in patients with T1D.

**Materials and methods:**

Data provided by the First People’s Hospital of Yunnan Province, 13 patients with T1D, were recruited and provided with an intervention for at least two weeks. All patients were asked to wear a continuous glucose monitoring (CGM) device under free-living conditions during the study period. To tackle the challenge of incomplete data from wearable devices for CGM measurements, the GAIN method was used in this paper to achieve a more rational interpolation process. In this study, patients’ PPGRs were calculated, and a LightGBM prediction model was constructed based on a Bayesian hyperparameter optimisation algorithm and a random search algorithm, which integrated glucose measurement, insulin dose, dietary nutrient content, blood measurement and anthropometry as inputs.

**Results:**

The experimental outcomes revealed that the PPGR prediction model presented in this paper demonstrated superior accuracy (R=0.63) compared to both the carbohydrate content only model (R=0.14) and the baseline model emulating the standard of care for insulin administration (R=0.43). In addition, the interpretation of the model using the SHAP method showed that blood glucose levels at meals and blood glucose trends 30 minutes before meals were the most important features of the model.

**Conclusion:**

The proposed model offers a heightened precision in predicting PPGR in patients with T1D, so it can better guide the diet plan and insulin intake dose of patients with T1D.

## 1 Introduction

Diabetes is a metabolic disorder that causes abnormal regulation of blood glucose, if not managed properly, it can lead to short- and long-term health complications and even death (1). At the present time, there is no cure for diabetes. However, self-management of the disease, particularly keeping blood glucose levels within the recommended range, is central to treatment. This includes actively tracking blood glucose levels, managing physical activity, diet and insulin intake (2).

The postprandial glycaemic response (PPGR) has a very important impact on overall glycaemic control and is a difficult aspect of T1D glycaemic control (3). Optimally dosing insulin at each meal presents a significant challenge in disease management. Accurately determining the appropriate insulin dosage is critical for regulating blood glucose levels and avoiding both hyperglycaemia and hypoglycaemia (4). In previous studies, researchers have typically used carbohydrates and insulin doses to predict blood glucose concentrations. However, the predictive accuracy of these models varies from person to person (5, 6). In addition to the nutritional content characteristics of the food consumed, changes in blood glucose may also arise from preprandial blood glucose, the patient’s lifestyle, and their clinical data. Mendes et al. (7) tested the efficacy of a prediction model for personalised postprandial glycaemic response developed using an Israeli cohort, which took into account characteristics such as food composition, blood, and lifestyle when applied to individuals in the Midwestern U.S. The results of the study demonstrated that the precision prediction method was more accurate in predicting blood glucose levels than the traditional method, which relied solely on the energy and carbohydrates in food. Thus, the most successful strategy for controlling blood glucose concentrations depends on the characteristics of each individual.

Eating habits are strongly influenced by ethnicity and region. For example, the Chinese have a very complex diet (8). A large number of current postprandial glucose prediction models for type 1 diabetes are based on Western dietary structures. Due to the complexity and uniqueness of the dietary structure, postprandial glycaemic control in Chinese patients with T1D faces unique challenges.

Therefore, the aim of this study was to construct a personalised model for predicting PPGR applicable to patients with T1D by collecting data on insulin dose, nutrient content of diet and additional clinical indicators from 13 patients with T1D in Kunming, Yunnan Province, in order to better guide the dietary plan as well as the dose of insulin intake in patients with T1D.

## 2 Materials and methods

### 2.1 Research object

This study used data provided by the First People’s Hospital of Yunnan Province for the period from September 2023 to January 2024. Thirteen patients with T1D (10 females and 3 males) were recruited in Kunming, Yunnan Province, and an intervention lasting at least two weeks was provided to each patient. Following were the criteria for inclusion (1): aged 18 years or older. (2) Diagnosed with diabetes for more than 1 year. Participation in the study was excluded if the participant was suffering from active inflammatory, neoplastic disease, pregnancy or a history of antibiotic use in the three months before participation in the study, chemotherapy or radiotherapy in the past 2 months, chronic gastrointestinal disease, and chronic anaemia.

During the study period, all participating patients agreed to wear the SIBIONICS GS1 CGM continuous glucose monitoring device, which uses a subcutaneous sensor to measure blood glucose levels at five-minute intervals, under free-living conditions. The SIBIONICS GS1 Continuous Glucose Monitoring (CGM) System is a 14-day calibration-free RT-CGM that supports data sharing with caregivers and seamlessly integrates with the advanced ProView Remote Access Platform, enabling healthcare providers to monitor patients remotely. Clinical evaluations and user feedback have demonstrated excellent accuracy, with the GS1 CGM achieving a Mean Absolute Relative Difference (MARD) of 8.83%, a key measure of glucose monitor accuracy (lower MARD values indicate higher accuracy). In addition, the GS1 CGM has been tested in a variety of environments, including with over 1,600 hospitals, and has been used by over 600,000 users (9, 10).

Prior to wearing the continuous glucose monitoring device, medical staff collected comprehensive information from each patient, including anthropometric measurements (e.g., height, weight), a set of blood tests, and lifestyle and basic information questionnaires (gender, age, etc.). Patients were requested to adhere to their usual daily routines and dietary patterns, reporting their dietary intake for breakfast, lunch and dinner to physician on a daily basis in real-time. The weight of each meal was weighed by the patients themselves and then registered by the physician, and a mobile app - Sugar Sugar Circle’s food bank of foods was used to measure carbohydrate, protein and fat content. Sugar Sugar Circle is a mobile app for blood sugar self-management and peer support for people with type 1 diabetes. It provides a food bank of up to more than 300,000 food items, which is very much in line with Chinese dietary habits, and allows for quick access to nutrient information for the food you want to find, as well as quick calculation of nutrient content using a weight scale. The physician must accurately document the specific nutritional components and timing details of patients’ meals. Reported meal times were rounded to the nearest 5-minute interval. To improve compliance, patients were told that accurate recording was essential to obtain an accurate analysis of the PPGR of foods. Insulin was manually infused by the physicians before the patients’ meals and the exact insulin dose was recorded.

The following filtering measures were applied to all meals recorded in this study: 1) To mitigate the potential impact of neighbouring meals and their antecedent insulin dosages, other meals recorded within 90 minutes were excluded from the analysis. Many studies have shown that the effects of mealtime insulin on insulin levels in subjects usually gradually return to basal levels within approximately 90-120 minutes after eating a meal. For example, the study by Hayashi et al. (11) details that in the oral glucose tolerance test (OGTT), insulin concentrations typically peak 30 to 60 minutes after glucose intake and approach basal levels 90 to 120 minutes after the meal. Shankar et al. (12) used the Mixed Meal Tolerance Test (MMTT) to study insulin levels and showed that insulin levels returned to basal levels within 90 to 120 minutes after a meal. Therefore, to ensure that the effect observed was that of a single meal alone and not the result of multiple meals superimposed on each other, we chose 90 minutes as a threshold that would allow for a better separation of the effects of taking insulin between meals. 2) Incomplete meal records were deleted. 3) Records of meals with a carbohydrate content of greater than 200 grams were deleted. According to the recommendations of the Institute of Medicine of the National Academy of Sciences (13), carbohydrates should account for 45-65 per cent of total daily calories, so an intake of 200 grams of carbohydrates per meal is considered abnormally high, and these outliers can have an asymmetric effect on the overall analysis, leading to distorted results.

### 2.2 Data pre-processing

Dealing with missing data presents a significant obstacle in analysing information gathered from wearable devices, frequently stemming from incorrect or delayed usage. Statistical imputation, matrix decomposition, and machine learning algorithms are among the frequently used computational techniques for addressing the challenge of incomplete data. However, these approaches often fail to adequately capture the temporal fluctuations inherent in time series data, leading to occasional interpolation outcomes that may appear unreasonable (14).

**Figure 1:**
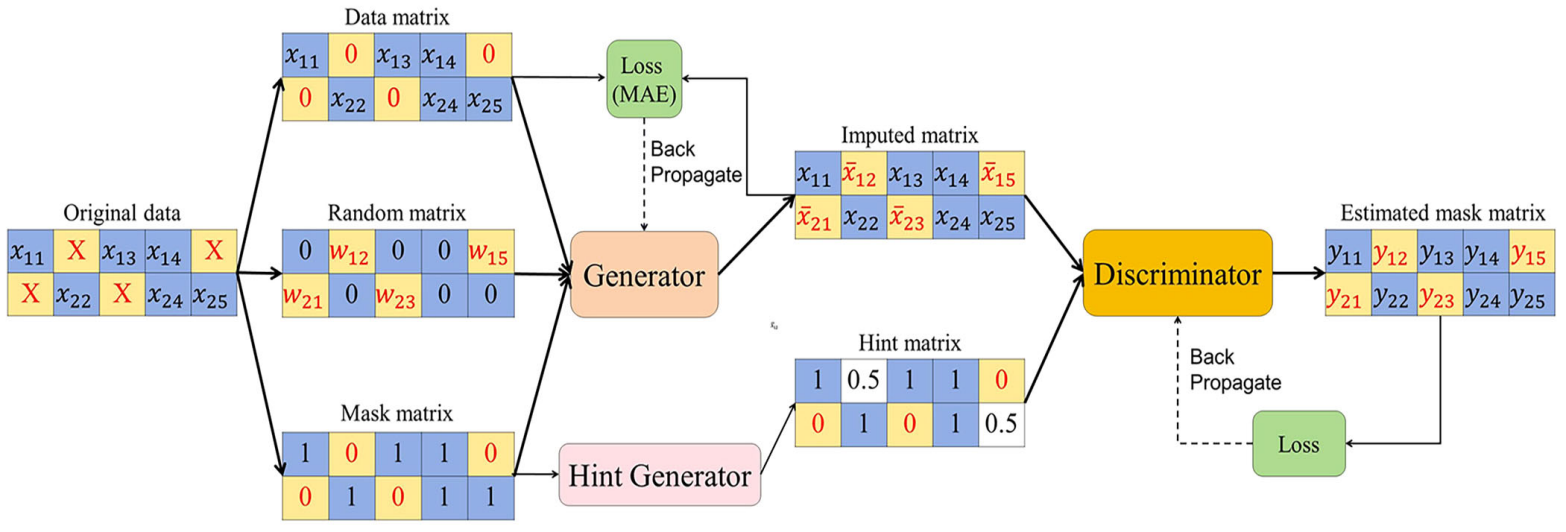
Process of missing data imputation using GAIN.

GAIN (Generative Adversarial Imputation Networks) is a generative adversarial network (GAN) approach for processing missing data (15).The GAIN framework consists of a generator and a discriminator. In GAIN, the generator fills the data and the discriminator distinguishes between real and generated data. The discriminator aims to minimize classification errors, while the generator seeks to maximize the discriminator’s error. Consequently, both networks undergo training through an adversarial process. To ensure that the adversarial training achieves the desired goal, GAIN assists the discriminator with a hint mechanism that ensures that the generator generates samples according to the distribution of the real data (**Figure 1**).

Generator: The generator G receives input consisting of a data matrix, a random matrix, and a mask matrix. The data matrix contains known data, but may also have missing values. The random matrix exclusively contains missing data and is populated with random values at the missing positions. The mask matrix is used to mark the positions of the missing values in the data matrix. Then, the generation process can be represented as follows:


$$\bar{X}=G\left(\tilde{X},M,\left(1-M\right)⊙Z\right)$$

where ⊙ represents the multiplication at the element level, $\bar{X}$ represents the output matrix, M represents the mask matrix, $\tilde{X}$ represents the data matrix, and Z represents the random matrix. This configuration closely resembles a typical GAN, with Z resembling the noise variables introduced in that structure.

Discriminator: In the GAIN framework, a discriminator D is introduced to continually counter the generator G. Nevertheless, in contrast to conventional GANs, the generator’s output comprises both genuine and spurious elements. The goal of the discriminator is not to identify the truth of the whole vector, but to identify which components of the vector are real and which are fake (i.e. interpolated).

Hint: The hint mechanism is intended to specify the positions of both the true and generated values, enhancing control over the direction of interpolation adjustments.

The Mean Absolute Error (MAE) criterion in this paper is adopted to assess the accuracy of the estimated values in relation to the actual values:


$$MAE=\underset{f}{\min}\sum_{\left(i,j\right)\epsilon M}\frac{\left|{\hat{x}}_{ij}-{\bar{x}}_{ij}\right|}{\left\|M\right\|}$$

### 2.3 Prediction of postprandial glycaemic response

In order to measure the effect of the meal on blood glucose, two metrics (PPGR and Glu_max_) were calculated in this study (16).

Firstly, according to the method of Zeevi et al. (17), the PPGR for every meal was computed by integrating meal times using CGM data and determining the incremental area under the curve (iAUC) of the blood glucose curve within a 2-hour postprandial window. The median of the blood glucose values during the first 30 minutes of the meal was taken as the initial blood glucose concentration. This initial concentration will be used as the reference value for calculating the incremental area under the curve. The final result of this procedure is the PPGR per meal based on the calculated incremental area under the curve and the initial blood glucose concentration:


$$PPGR=\frac{\sum_{i=1}^{n}\frac{h_{i}}{2}\cdot \left(y_{i-1}+y_{i}-2y_{0}\right)}{y_{0}}$$

where n represents the number of time points, $h_{i}$ represents the time interval between two adjacent time points, $y_{i-1}$ and $y_{i}$ are the blood glucose measurements at two adjacent time points, and $y_{0}$ represents the initial blood glucose concentration.

Second, the variance in blood glucose levels at mealtime and the maximum blood glucose level within 2 h after the meal (Glu_max_) was calculated. This metric was selected due to its reduced sensitivity to inaccuracies in patients’ logging times:


$$Glu_{\max}=\max_{i=1}^{n}y_{i}-y_{0}$$

In order to predict these two metrics (PPGR and Glu_max_), a LightGBM prediction model based on Bayesian hyperparameter optimisation algorithm combined with stochastic search algorithm was constructed in this paper. Model inputs consisted of 38 features in total, encompassing features such as meal composition and blood test outcomes, blood glucose measurements and insulin doses. 60% of the meals were utilized for training the model, while the remaining 40% were reserved for validation purposes.

The experiment was conducted on a computer with Windows 11 operating system. The simulation platform is Pycharm and is programmed using Python with sklearn, pandas and numpy libraries.

#### 2.3.1 LightGBM model

The primary concept behind GBDT (Gradient Boosting Decision Tree) is to iteratively train using a weak classifier (decision tree) to obtain an optimal model, while LightGBM optimises the traditional GBDT algorithm as follows (18): histogram algorithm, gradient-based one-sided gradient sampling (GOSS), exclusive feature bundling (EFB), and leaf-wise growth strategy with depth constraints.

The basic idea of the histogram algorithm is to first discretise the continuous floating-point eigenvalues intointegers, and at the same time construct a histogram with a width of. When traversing the data, statistics are accumulated in the histogram based on the discrete values as indexes, when traversing the data once, the histogram accumulates the required statistics and then traverses to find the optimal segmentation point based on the discrete values of the histogram.

The basic idea of GOSS (gradient-based one-side sampling) is to calculate the gradient of the samples and then keep only the samples with larger gradient. This reduces the number of trainings samples and improves the training efficiency while maintaining similar information. The set of samples for GOSS sampling is N , and the threshold of gradient is α , then the sampling process is as follows:


$$N=\left\{i|\left|n_{i}\right|>\alpha \right\}$$

where $n_{i}$ is the gradient of the sample i .

The basic idea of the EFB (exclusive feature bundling) algorithm is to reduce the number of features and improve the generalisation ability of the model by bundling the features and merging the highly correlated features into one feature group.

**Figure 2:**
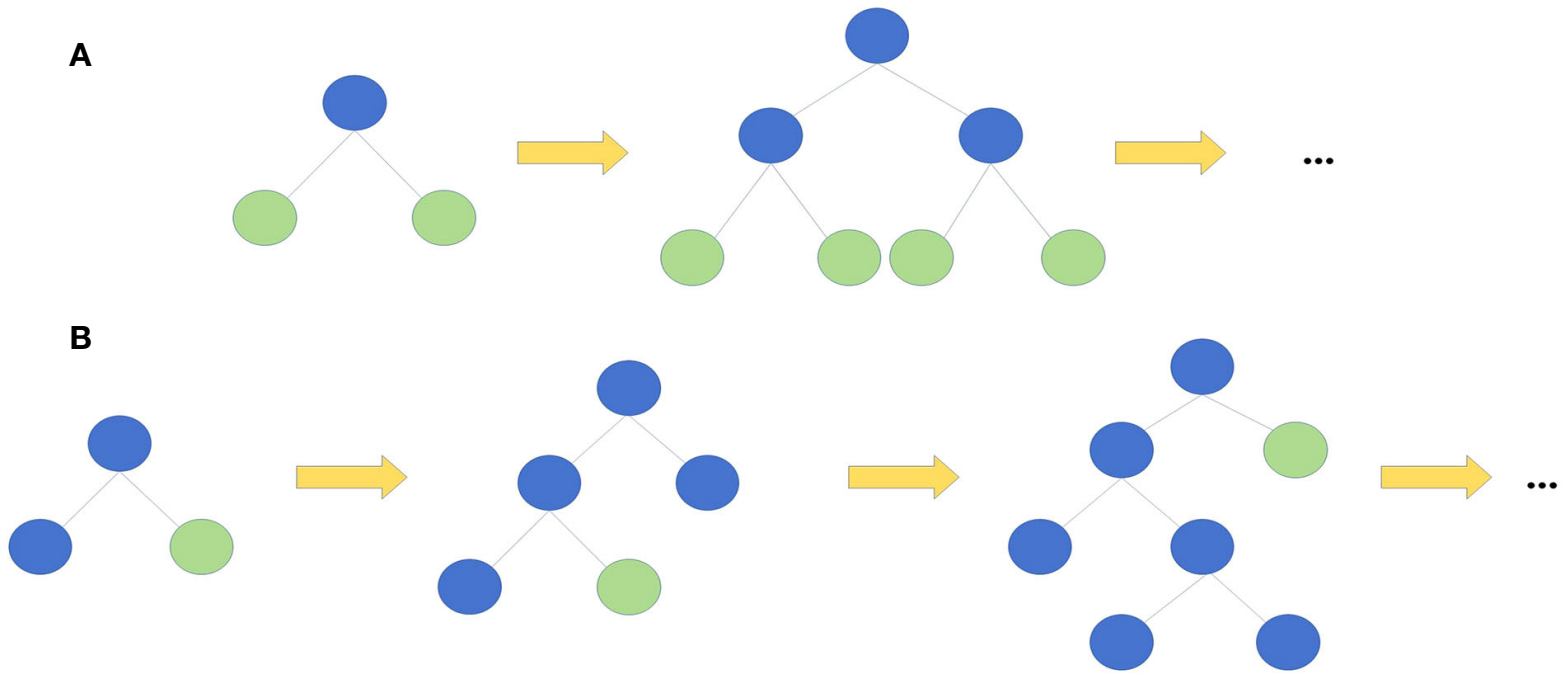
Two kinds of tree growth strategy. **(A)** Level-wise growth strategy **(B)** Leaf-wise growth strategy. ..., and so forth.

The leaf-wise algorithm with depth constraints aims to reduce the complexity of the model and improve the training efficiency by controlling the depth of the tree and the number of leaf nodes (**Figure 2**). LightGBM firstly divides the dataset into different histograms according to the range of values of the features. Such a division can speed up the training process because the histograms can replace the original data in decision tree learning, reducing memory and computation. During each tree growth, instead of splitting based on nodes, the tree is split based on leaf nodes to find the leaf node with the maximum splitting gain among all current leaf nodes. Such a leaf splitting strategy reduces the risk of overfitting and improves model generalisation.

In LightGBM, the objective function consists of two parts, one is a measure of the fit to the training data and the other is a measure of the model complexity to avoid overfitting. The objective function can be represented like this:


$$\begin{array}{l} Obj^{\left(k\right)}=\sum_{t=1}^{n}\;l\left(y_{t},y_{t}^{\left(k\right)}\right)+\sum_{t=1}^{T}\text{ Ω}\left(f_{t}\right)\\ =\sum_{t=1}^{n}\;l\left(y_{t},y_{t}^{\left(k-1\right)}+f_{k}\left(x_{t}\right)\right)+\sum_{t=1}^{T}\text{ Ω}\left(f_{t}\right) \end{array}$$

where, k indicates the overall count of iterations, n represents the quantity of training samples, $y_{t}$ is the true value of the t th training sample, $y_{t}^{\left(k\right)}$ is the predicted value of the t th training sample, $l\left(y_{t},y_{t}^{\left(k\right)}\right)$ is the loss function, $f_{k}\left(x_{t}\right)$ is the anticipated impact of the decision tree to the t th training sample $x_{t}$ in the k th iteration, and $\text{Ω}\left(f_{t}\right)$ is the regularisation term.

In each iteration, the goal of the model is to minimise the aggregate loss across all training samples by finding a new decision tree, while also considering the model’s complexity aimed at mitigating overfitting. When adding a new decision tree, the model considers a combination of loss functions and regularisation terms to minimise the loss of training data while maintaining the model’s ability to fit.

#### 2.3.2 Bayesian hyperparametric optimisation algorithm

In order to improve the accuracy of the LightGBM prediction model, in this paper, a Bayesian hyperparameter optimisation algorithm combined with a stochastic search algorithm is used to automatically search for the optimal parameter configurations of the model. Hyperopt is one of the Bayesian optimisation libraries in Python, which uses an optimisation algorithm called Tree Parzen Estimation (TPE) (19). The core idea of TPE is to use the information about the parameter combinations that have been explored to dynamically adjust the parameter search space for the next iteration, so that better hyperparameter combinations can be found within a limited number of iterations. By transforming the generative process that describes the configuration space X , the TPE model p(x|y) replaces the distribution of *a priori* configurations with non-parametric densities. Each iteration of TPE not only scales linearly according to the number of samples, but also optimises the number of dimensions in the parameter space by maintaining the ordering of the observed variables.


$$p\left(x|y\right)={\{}_{g\left(x\right)\;if\;y\;\geq y^{*}}^{\ell \left(x\right)\;if\;y\;<\;y^{*}}$$

where ℓ(x) and g(x) denote the observations and the rest of the observations.

When using Hyperopt for hyperparameter optimisation, a new approach is used where the data is first randomly sampled. The core idea of this approach is that since the sample is representative of the entire population, a sample can be used instead of the entire training dataset, and then Hyperopt is used to generate the optimal hyperparameters for LightGBM, an approach that greatly reduces the execution time required to generate the optimal hyperparameters (20).

Bayesian hyperparameter optimisation using Hyperopt is performed by combining a random search algorithm with a set of hyperparameters randomly selected from the search space to try in each iteration. By randomly sampling a set of hyperparameters in the hyperparameter space, their performance is evaluated and then the best performing set of hyperparameters is selected. This helps to avoid falling into a local optimal solution, thus enabling a more global search.

The set of hyperparameters for this study includes the following: learning_rate is used to control the magnitude of the update at each step, a smaller learning rate makes the model converge more slowly but may result in better generalisation performance; n_estimators specifies the number of weak learners, i.e. the number of decision trees to be trained; max_depth is the maximum depth of each tree, which controls the tree’s complexity, a larger depth may lead to model overfitting; colsample_bytree is the proportion of features used in each tree, which controls the proportion of features randomly selected in constructing each tree and prevents overfitting; min_child_samples is the minimum number of samples required for each leaf node, which prevents overfitting; num_leaves is the number of maximum number of leaf nodes per tree; subsample is the proportion of samples used per tree, which controls the proportion of samples randomly selected during training of each tree and prevents overfitting.

#### 2.3.3 LightGBM prediction model based on Bayesian hyperparameter optimisation algorithm combined with stochastic search algorithm

**Figure 3:**
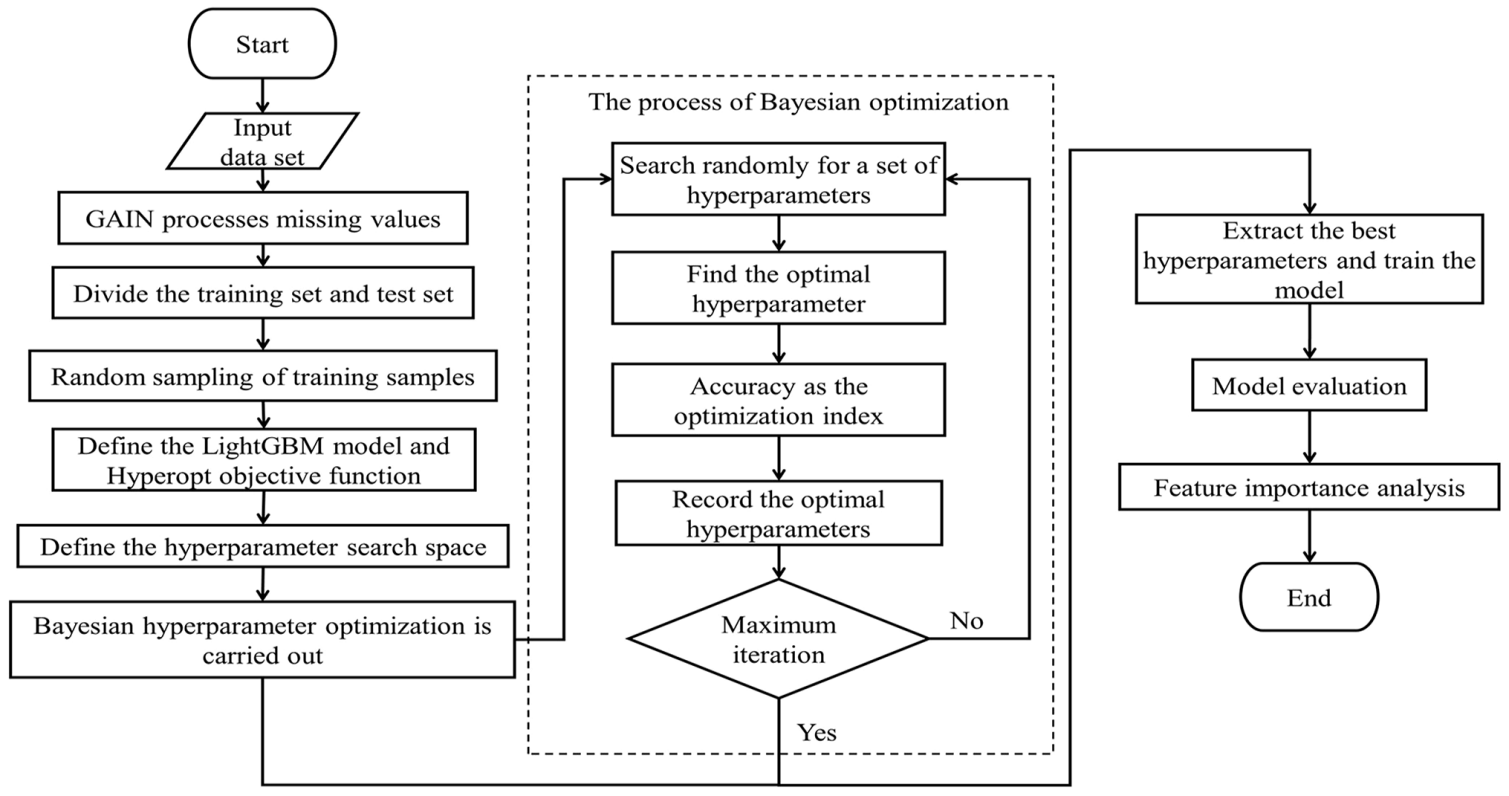
Experiment flow chart.

In this paper, a Bayesian hyperparameter optimisation algorithm combined with a stochastic search algorithm is used to optimise the LightGBM model and develop a model to predict PPGR in patients with T1D. The specific experimental procedure is shown in **Figure 3**.

Taken together, as described in Sections 2.3.2 and 2.3.2, the LightGBM model combining Bayesian hyperparameter optimisation algorithm and stochastic search algorithm has the advantages of high efficiency, adaptive and global optimisation, which can effectively improve the performance and generalisation of the model, and it can predict the patients’ PPGRs more efficiently and accurately.

### 2.4 Feature attributions

To further understand the factors that influence model predictions, in this study, Shapley Additive exPlanations (SHAP) is employed to achieve model interpretability (21–23).

Shapley Additive exPlanations (SHAP) is a method for interpreting machine learning model predictions based on the concept of Shapley values from cooperative game theory. In machine learning, the SHAP method provides each feature with its contribution to the model prediction by applying this concept to the interaction between features. This method of interpretation not only provides interpretability for model predictions, but also can help understand the logic behind the model and the interactions between features. The ability to correctly interpret the predictive model outputs is important in providing insights into how to improve the model, as well as an understanding of the process being modelled.

SHAP has a wide range of applications for interpreting various types of machine learning models, including decision trees, neural networks, and integrated models, etc. SHAP estimates the contribution of each feature by ranking and combining a subset of features, and this feature-interaction-based interpretation approach allows SHAP to reveal the complexity of model predictions and help users understand the model’s decision-making process. In clinical applications, these interpretations provide important information to guide doctor-patient discussions when the model categorises patients as being at high risk of certain adverse outcomes (24). Therefore, the SHAP method is important in interpreting machine learning models and has been widely used and recognized in practical applications.

In this paper, SHAP was used to interpret the PPGR prediction model in order to reveal important features affecting postprandial glucose elevation in patients with T1D, with the aim of providing more tailored guidance to healthcare professionals to help patients with T1D to improve their lifestyle habits and optimize the dose of insulin intake.

## 3 Experiments and results

### 3.1 Study population

A total of 13 patients with T1D (10 females and 3 males) were recruited into this study between September 2023 and January 2024, and a total of 867 meals were recorded during the study period, with a final sample of 826 usable meals selected for modelling. Of these, the mean age was 3810 years (median 35 years, interquartile range [IQR] 32-46 years), the mean BMI was 212.1 kg/m^2^ (median 21.3 years, interquartile range [IQR] 20-22 kg/m^2^), and the mean HbA1c level was 8.08%2.26% (see **Table 1** for an analysis of all the blood test results).

**Table 1:** Blood test results.

Blood test result	Mean	Standard Deviation
HbA1c (%)	8.08	2.26
Creatinine (umol/l)	60.53	12.64
Sodium (mmol/l)	137.33	0.55
Potassium (mmol/l)	4.19	0.24
Serum chloride (mmol/l)	105.57	1.52
Calcium (mmol/l)	2.27	0.08
Total bilirubin (umol/l)	12.71	3.16
Uric acid (umol/l)	296.31	56.32
ALT (u/l)	16.86	6.55
AST (u/l)	19.03	5.74
ALP (u/l)	74.16	21.05
Total protein(g/l)	71.12	6.54
ALB (g/l)	43.05	2.57
Cholesterol (mmol/L)	9.11	10.79
Triglycerides (mmol/L)	1.05	0.54
HDL (mmol/L)	1.55	0.31
LDL (mmol/L)	2.74	1.24
TSH (mIU/L)	3.69	1.89
Fasting C peptide levels (nmol/L)	0.02	0.02

**Figure 4:**
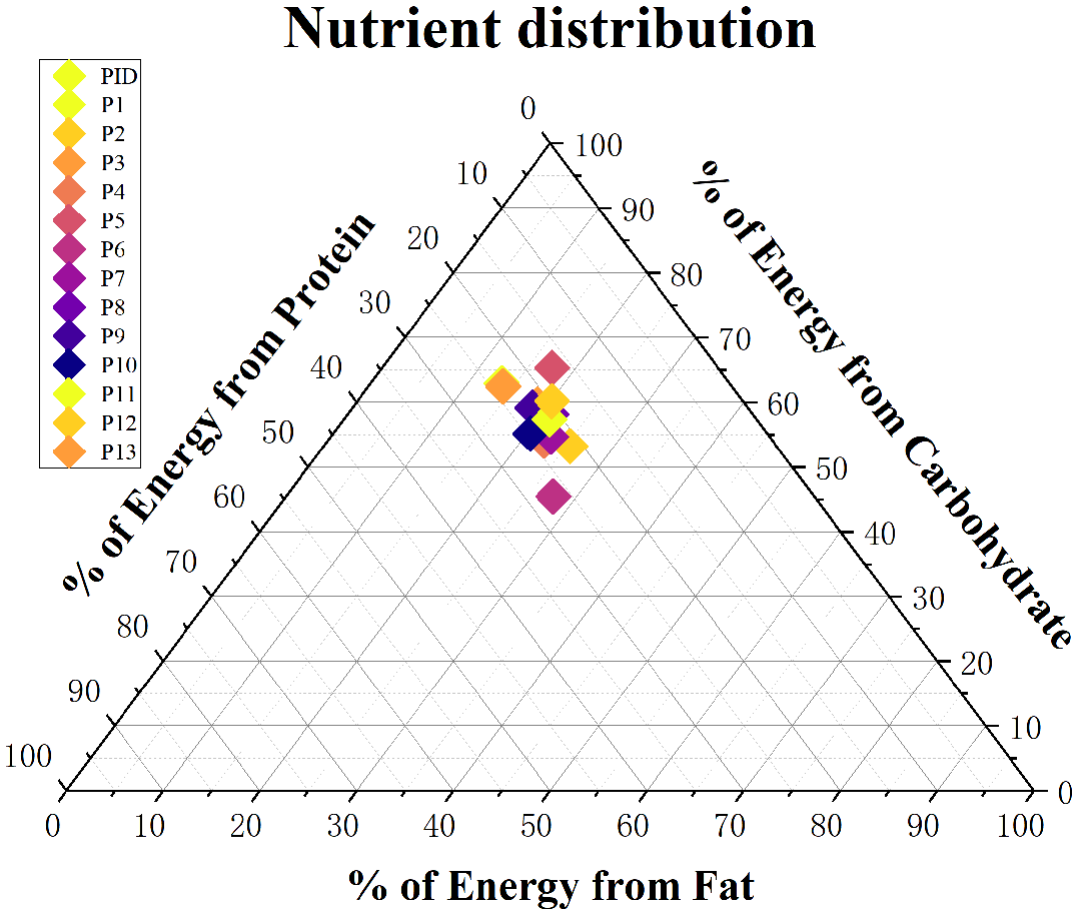
Distribution of the mean daily macronutrient proportions derived from the total energy intake of participants in the cohort (each participant represented by a single point).

In order to be able to visualize the patients’ dietary habits more closely, the distribution of macronutrient intake in total energy intake was analysed in this study (**Figure 4**). The average carbohydrate, fat and protein consumption was 53.611.5g, 19.15.9g and 20.74.4g, respectively.

### 3.2 GAIN processing results

In this paper, the GAIN algorithm was applied to the processing of missing values in patients’ continuous glucose data (CGM). In order to validate the effectiveness of the GAIN model used to process missing data, in this study, all instances of missing data in the original dataset were removed to obtain a intact validation set. For the validation set, ten percent of the data were randomly chosen to serve as missing values. The MAE between the generated interpolated values and the true dataset values was employed as the metric. In this paper we compared the results of three data interpolation methods for the blood sugar data processing, including GAIN, K-Nearest Neighbour (KNN) interpolation and Linear interpolation.

The experimental results demonstrate that the GAIN model achieved the optimal imputation performance for CGM data, with a mean absolute error (MAE) of 16.11 mg/dL, significantly lower than the KNN interpolation algorithm’s 20.16 mg/dL and the linear interpolation algorithm’s 19.8 mg/dL. This indicates that the GAIN model outperforms the other two traditional methods in imputing time-series blood glucose data.

### 3.3 Predicting the glycaemic response to a realistic diet

In this paper, a LightGBM prediction model was constructed based on a Bayesian hyperparameter optimization algorithm combined with a stochastic search algorithm. Additionally, the ability of the model to predict PPGR and Glu_max_ was assessed by calculating the Pearson correlation coefficients between the predicted and observed values:


$$p=\frac{\mathrm{cov}\left(X,\bar{X}\right)}{\sigma_{X}\sigma_{\bar{X}}}$$

where $\bar{X}$ represents the actual observed values, $\bar{X}$ represents the predicted values, $\mathrm{cov}\left(X,\bar{X}\right)$ is the covariance of X and $\bar{X}$ , and $\sigma_{x}$ and $\sigma_{\bar{x}}$ are the standard deviations of X and $\bar{X}$ , respectively.

In order to validate the accuracy rate of the model, compared to other models using only carbohydrates as well as insulin dose to predict PPGR, in this study, a LightGBM prediction model that included the following three Bayesian hyperparametric optimisation algorithms combined with stochastic search algorithms was constructed: 1) a model based on carbohydrate content only: only one feature of carbohydrates in the food was used as an input. 2) An insulin administration baseline model: carbohydrate content, pre-meal insulin dose, and blood glucose level at the time of the meal were used as input features. 3) A full model: incorporating as inputs all of the information gathered from the patients over the duration of the study, encompassing features such as meal composition and blood test outcomes, blood glucose measurements, and insulin dosage (for features included in the model see **Table 2**).

**Table 2:** Features included in the model.

Category	Features
Meal content	Calorie, proteins, fats, carbohydrates, carbohydrate/fat ratio, carbohydrate/proteins ratio
Blood tests results	HbA1c,creatinine,sodium,potassium,serum chloride,calcium,total bilirubin,uric acid,alanine transaminase(ALT),aspartate transaminase(AST),total protein,alkaline phosphatase(ALP),cholesterol,triglycerides,HDL,LDL,thyrotropin,fasting C-peptide levels,glucose),alkaline phosphatase(ALP),total protein,albumin,cholesterol,triglycerides,HDL,LDL,thyrotropin,fasting C-peptide levels,glucose
Anthropometric measurements	Weight, height, waist and hips circumference, BMI
Survey- derived features	Age, gender
CGM-derived features	Glucose value at meal initiation,glucose trends calculated by subtraction of glucose value at meal initiation from The glucose values at 30, 60 and 120 minutes before the commencement of the meal.
Insulin	High dose of insulin before meal,4 hours basal insulin

**Figure 5:**
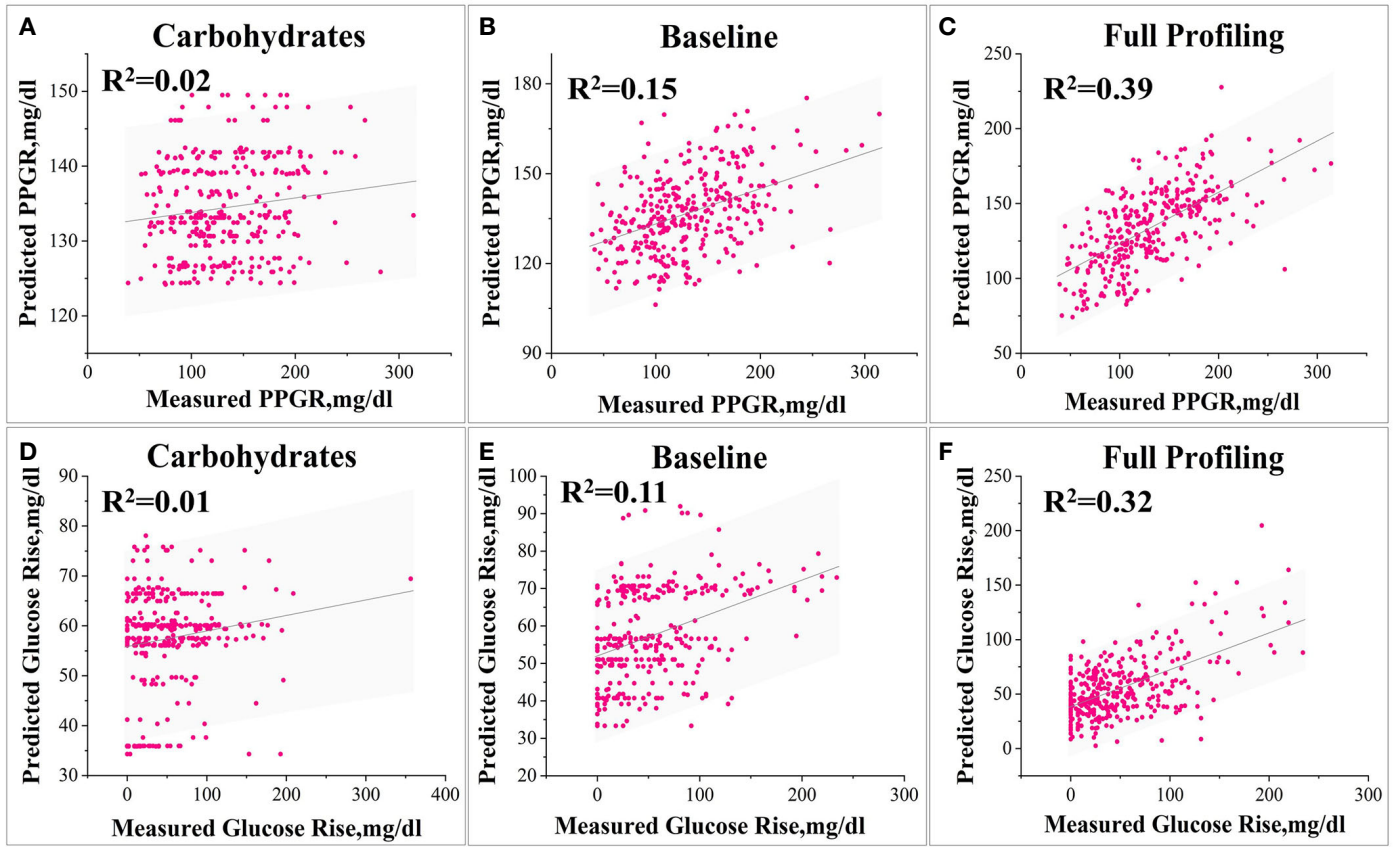
Various model predictions of PPGR and Glu_max_. 1) Models for Predicting PPGR: Model **(A)** based solely on postprandial carbohydrate content, Baseline model simulating insulin administration **(B)**, and Model **(C)** utilizing all features. 2) Models for Predicting Glu_max_: Model **(A)** based solely on postprandial carbohydrate content, Baseline model simulating insulin administration **(B)**, and Model **(C)** utilizing all features.

In the prediction of PPGR, the model relying solely on carbohydrate content demonstrates a relatively low correlation (R=0.14, **Figure 5A**) of its predictions with observed PPGRs and explains only about 2% of the variance in glycaemic response. The insulin administration baseline model performs better (**Figure 5B**), with a correlation of its predictions with the observed PPGRs is 0.43 (R=0.43, P<10^-10^) and explains 15% of the variance in glycaemic response. The full model integrating glucose measurements, insulin dose, meal content, and blood characteristics achieves a significantly higher correlation (R=0.63, P<10^-10^) and the explained variance increases to 39% (**Figure 5C**).

After parameter optimisation of the LightGBM model using a Bayesian optimisation algorithm combined with a stochastic search algorithm, the optimal hyperparameter settings for the complete model are obtained as follows: learning_rate is 0.009, n_estimators is 345, max_depth is 5, colsample_bytree is 0.75, min_ child_samples is 2, num_leaves is 36, and subsample is 0.69.

Similarly, for Glu_max_ predictions, the model relying solely on carbohydrate content has a correlation that is relatively low (R=0.15) (**Figure 5D**), the baseline model performs better (R=0.38, P<10^-10^) (**Figure 5E**), and the full model has a significantly higher correlation (R=0.58, P<10^-10^) (**Figure 5F**).

### 3.4 Characteristic attribution results

**Figure 6:**
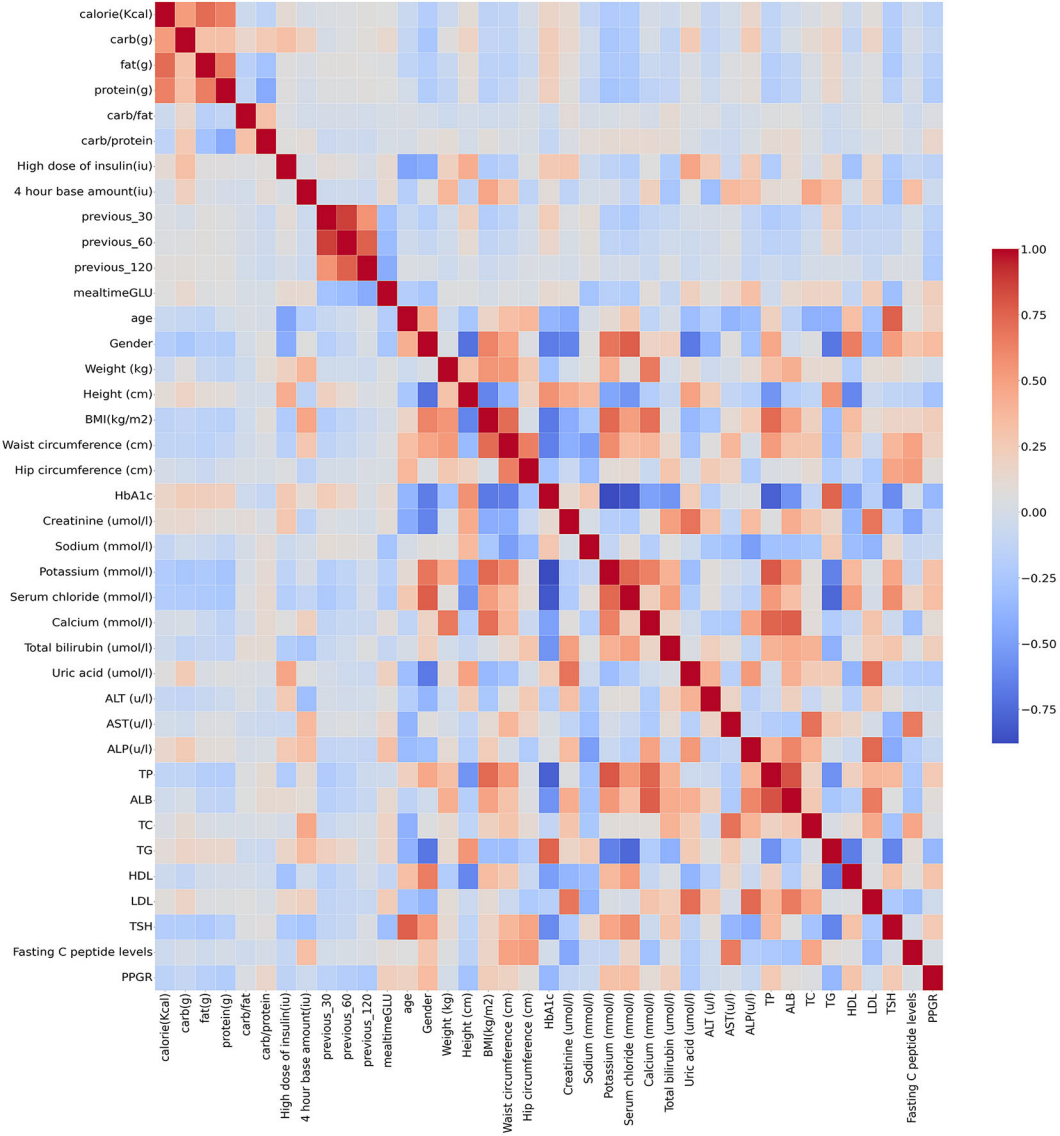
Correlation of clinical parameters (red for positive correlation, blue for negative correlation).

In order to clearly observe the relationship between the various parameters, a heat map was used. The heatmap provided a clearer visualisation of the linear relationship between the various features and PPGRs (**Figure 6**). From the heat map, it can be seen that the correlation coefficients of ALT, AST and TSH with PPGR seem to be close to 0, i.e., there is almost no linear relationship, whereas 4 hour base amount, and high dose of insulin have a strong positive correlation with blood glucose level at meal time.

**Figure 7:**
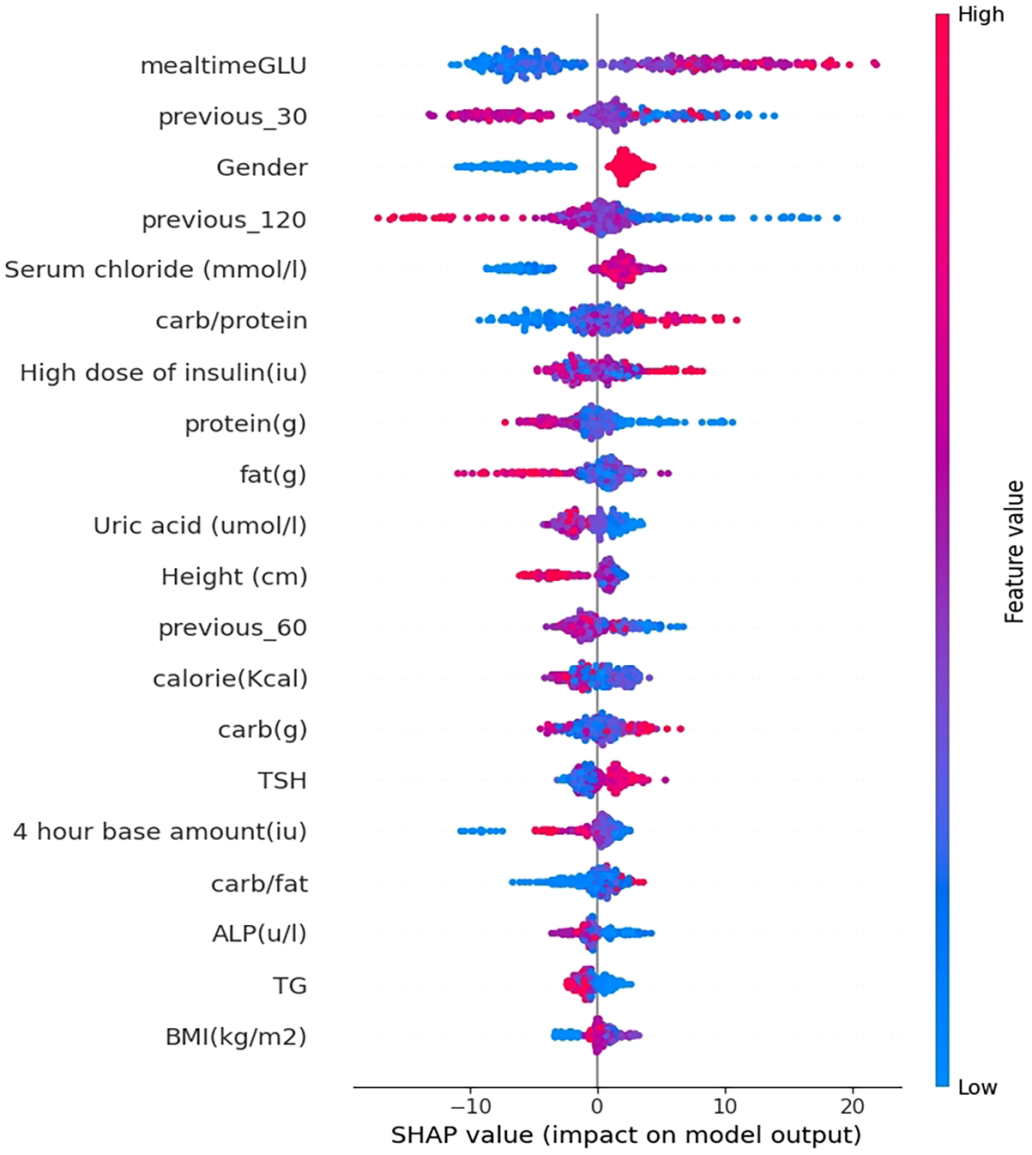
Interpretation of the prediction model.

To further understand the factors affecting the model predictions, Shapley Additive exPlanations (SHAP) was used in this paper to achieve model interpretability. The results of using SHAP to assess feature importance are shown in **Figure 7**. The illustration portrays the influence of the top 20 most substantial features (arranged in descending order from top to bottom) on predicting PPGR for a particular data point within the test set. Each feature’s effect on the prediction (SHAP value) is displayed on the scale. The distance from zero (indicated by a gray vertical line) indicates the magnitude of the feature’s influence on the model. The colours represent the feature’s value at each point, spanning from below-average (blue) through average (purple) to above average (red). For the most important feature blood glucose level at meals, it is clear that lower blood glucose levels at meals lead to significantly lower PPGR values.

It follows that the model’s most impactful features with the highest mean absolute SHAP values include blood glucose level at meal time, blood glucose trend 30 minutes before the meal, gender, serum chloride, blood glucose trend 120 minutes before the meal, carbohydrate-to-protein ratio, protein content, calorie content, carbohydrate content, and fat content (**Figure 7**).

## 4 Discussions

In this paper, a personalised postprandial glycaemic response prediction model for patients with T1D is proposed, using LightGBM based on a Bayesian hyperparametric optimisation algorithm combined with a stochastic search algorithm. The input features of the model include features such as meal composition and blood test outcomes, glycaemic measurements and insulin dose.

Postprandial glycaemic response (PPGR) is an important indicator of the effectiveness of glycaemic control and glucose metabolism in all types of diabetic patients. Clinical trials have shown the importance of keeping postprandial glucose within the normal range (25, 26). In recent years, the increased use of continuous glucose monitors (CGMs) among diabetic patients (27) has radically improved the application of predicting postprandial glucose responses. However, modelling different individuals remains a challenge. For example, Kezhi Li et al. (28) utilized GluNet, a personalized deep neural network framework, to predict the short-term (30-60 minutes) probability distribution of future CGM values in T1D subjects using historical data, including glucose measurements, dietary information, insulin dosage, and other factors. In 2017, KOREM et al. (29) conducted a randomised crossover trial in which 20 healthy subjects consumed two types of bread to compare their PPGRs and other clinical metrics. After careful examination of individual responses, it was found that there were significant differences in PPGRs between individuals after bread consumption. Models that incorporate individual-specific factors have been shown to be more effective in predicting an individual’s PPGR than traditional methods. These personalised models rely on key variables, including anthropometric measurements, dietary intake, etc., to accurately predict PPGR.

There are also several studies of PPGR prediction models that include CGM-related characteristics, gut microbiome characteristics of individuals, anthropometrics, and dietary macronutrients, as shown in **Table 3**.

**Table 3:** Summary statistics from previous studies (correlation coefficient R).

Reference	Statistics R	Cohort
Shilo et al., 2022 (16)	R=0.59, Full model	A cohort of Type 1 diabetes patients from Israel
Pustozerov et al., 2020 (30)	R=0.53, Full model	A cohort of patients with gestational diabetes
Zeevi et al., 2015 (17)	R=0.7, Full model	A cohort of non-diabetic adults from Israel
Mendes-Soares et al., 2019b (31)	R=0.62, Full model	A non-diabetic cohort from the Midwest
Tily et al., 2022 (32)	R=0.77, Full model	The U.S. Health Cohort, of which 73 per cent were Caucasian

In the above study, Shilo et al. (16) developed a model for predicting PPGR in patients with T1D using a cohort of Israeli T1D patients and the inputs to the model also included microbiome profiles. Pustozerov et al. (30) used data from patients with gestational diabetes to construct a PPGR model. Whereas Zeevi et al. (17), Mendes-Soares et al. (31) and Tily et al. (32) all used healthy cohorts. Thus, the generally higher correlation between PPGRs obtained from CGM extracted from healthy individuals and PPGRs obtained from blood tests also suggests that predicting glycaemic response to diet is more challenging in patients with T1D than in healthy individuals, as patients with T1D have higher glycaemic variability.

These studies are all based on Western dietary structures, however, Chinese dietary habits are very complex, so more accurate postprandial glycaemic response (PPGR) prediction models are needed to guide postprandial glycaemic control in Chinese patients with T1D. In this paper, data from 13 patients with T1D in Kunming City, Yunnan Province are collected, provided by the First People’s Hospital of Yunnan Province, and we developed a personalised PPGR prediction model for patients with T1D, using LightGBM based on Bayesian hyperparameter optimisation algorithm combined with a stochastic search algorithm to construct the model. The input features of the model include features such as meal composition and blood test outcomes, blood glucose measurements and insulin doses. The experimental results show that the correlation (R=0.63) between the predictions of the model in this paper and the observed PPGR is better than that of the Shilo et al. (16) ‘s model (R=0.59), and that the model developed here does not necessitate microbiome data as input, enhancing its accessibility for clinical application. In the prediction of both PPGR and Glumax, the proposed model also significantly outperforms the traditional model relying solely on carbohydrate content in food and the baseline model simulating the current standard of care for insulin administration.

In addition, although the advent of continuous glucose monitors (CGMs) in recent years has significantly enhanced the application of CGMs in glucose prediction by providing a large amount of time-series data through real-time monitoring of blood glucose levels, incomplete monitoring data may occur due to factors such as inappropriate or untimely wearing patterns and sensor malfunctions. These missing data may affect the accuracy and stability of the prediction model. In this study, in order to fill the missing values in the blood glucose data more rationally, the GAIN algorithm was used, which has a great advantage in capturing the temporal variations of time series data. And from the results of the study, GAIN does have higher accuracy than traditional methods in the processing of blood glucose data.

In this study, it reveals the drivers of postprandial glucose elevation in patients with T1D by analysing the factors influencing the prediction model using SHAP. From the results of the study, the most influential features include blood glucose levels at the time of the meal, blood glucose trends 30 minutes before the meal, and carbohydrate to protein ratio. These results show that features related to CGM data have the greatest impact on the model, for example, the blood glucose level at mealtime, the blood glucose trend 30 minutes before meal and other features rank highly, followed by features related to dietary nutrient content. In this cohort, the lower the blood glucose level at mealtime and the lower the carbohydrate intake, the better the blood glucose control. These results are in good agreement with the results reported in the study by Shilo et al. (16).

Gender is also an important influencing factor in this study. In 2019, González-Rodríguez et al. (33) have demonstrated that the effects of dietary nutrients on postprandial glycaemic responses were different in women compared to men in a non-diabetic population. From the results in this study, it appears that gender characteristics also have an effect on postprandial glycaemic response in patients with T1D, but this conclusion may also be affected by the small sample size of the data and the uneven ratio of male to female patients.

In this study there has several limitations. Firstly, inaccuracies in patient self-reporting of dietary intake may affect the ability to predict postprandial glycaemic response. Secondly, because accurate dietary intake data are difficult to collect, the sample size of data in this paper is small and not representative of a broader population, and better predictions could have been obtained with more high-quality clinical data to train the model. Finally, although the PPGR prediction model proposed in this paper has a high level of accuracy, there is still potential for enhancement. For example, the inclusion of microbiome data and a detailed assessment of physical activity does increase costs, but may also improve the accuracy of the predictions.

## 5 Conclusions

In this study, a personalised PPGR prediction model for patients with T1D is proposed. For the model, glucose measurements, insulin dose, dietary content, blood measurements, and anthropometrics are integrated, and it is substantially superior to traditional models that rely solely on the amount of carbohydrates in food and baseline models that simulate the current standard of care for insulin administration. The proposed model could accurately predict postprandial glycaemic response in patients with T1D, and it maybe better guide patient dietary planning as well as insulin intake dosage. Furthermore, the proposed model can be further implemented within closed-loop systems, personalized decision support systems, and alert systems to mitigate anticipated hyperglycaemic and hypoglycaemic events in patients with Type 1 Diabetes (T1D). Additionally, the model can tailor dietary nutritional plans for T1D patients based on anticipated hypoglycaemic responses. In summary, the model represents a meaningful step forward in improving postprandial glycaemic control in T1D patients, providing direction for future research and development in personalized diabetes care.

## Data availability statement

The original contributions presented in the study are included in the article/supplementary material. Further inquiries can be directed to the corresponding authors.

## Ethics statement

The studies involving humans were approved by the Medical Ethics Committee of the First People’s Hospital of Yunnan Province. The studies were conducted in accordance with the local legislation and institutional requirements. The participants provided their written informed consent to participate in this study.

## Author contributions

XX: Conceptualization, Formal analysis, Project administration, Validation, Supervision, Writing – review & editing. YX: Methodology, Software, Visualization, Writing – original draft, Writing – review & editing. YC: Conceptualization, Data curation, Investigation, Project administration, Validation, Writing – review & editing. JH: Funding acquisition, Supervision, Writing – review & editing. HS: Funding acquisition, Resources, Validation, Writing – review & editing.

## References

[1] Harding JL Pavkov ME Magliano DJ Shaw JE Gregg EW. Global trends in diabetes complications: a review of current evidence. Diabetologia. (2019) 62:3–16. doi: 10.1007/s00125-018-4711-2 30171279

[2] Association AD. 5. Lifestyle management: Standards of medical care in diabetes—2019. Diabetes Care. (2018) 42:S46–60. doi: 10.2337/dc19-S005 30559231

[3] Ceriello A Colagiuri S. International diabetes federation guideline for management of postmeal glucose: a review of recommendations. Diabetic Med. (2008) 25:1151–6. doi: 10.1111/j.1464-5491.2008.02565.x PMC2701558 19046192

[4] Nathan DM Genuth S Lachin J Cleary P Crofford O Davis M. The effect of intensive treatment of diabetes on the development and progression of long-term complications in insulin-dependent diabetes mellitus. New Engl J Med. (1993) 329:977–86. doi: 10.1056/NEJM199309303291401 8366922

[5] Bell KJ Toschi E Steil GM Wolpert HA. Optimized mealtime insulin dosing for fat and protein in type 1 diabetes: Application of a model-based approach to derive insulin doses for open-loop diabetes management. Diabetes Care. (2016) 39:1631–4. doi: 10.2337/dc15-2855 27388474

[6] Wolpert HA Atakov-Castillo A Smith SA Steil GM. Dietary fat acutely increases glucose concentrations and insulin requirements in patients with type 1 diabetes implications for carbohydrate-based bolus dose calculation and intensive diabetes management. Diabetes Care. (2013) 36:810–6. doi: 10.2337/dc12-0092 PMC3609492 23193216

[7] Mendes-Soares H Raveh-Sadka T Azulay S Ben-Shlomo Y Cohen Y Ofek T. Model of personalized postprandial glycemic response to food developed for an Israeli cohort predicts responses in midwestern american individuals. Am J Clin Nutr. (2019) 110:63–75. doi: 10.1093/ajcn/nqz028 31095300 PMC6599737

[8] Mora N Golden SH. Middle eastern, and latino patients with type 2 diabetes: a review of current literature and future directions. Curr Diabetes Rep. (2017) 17:1–12. doi: 10.1007/s11892-017-0952-6 29063419

[9] Yan L Li Q Guan Q Han M Zhao Y Fang J. Evaluation of the performance and usability of a novel continuous glucose monitoring system. Int J Diabetes Developing Countries. (2023) 43:551–8. doi: 10.1007/s13410-022-01112-0

[10] Kesavadev J Saboo B Chawla M Parikh R Sahay R Joshi SR. The historical evolution of continuous glucose monitoring - the story of 25 years. Int J Diabetes Technology. (2023) 2:129–36. doi: 10.4103/ijdt.ijdt_16_24

[11] Hayashi T Boyko EJ Sato KK McNeely MJ Leonetti DL Kahn SE. Patterns of insulin concentration during the ogtt predict the risk of type 2 diabetes in Japanese americans. Diabetes Care. (2013) 36:1229–35. doi: 10.2337/dc12-0246 PMC3631850 23275353

[12] Shankar SS Vella A Raymond RH Staten MA Calle RA Bergman RN. Standardized mixed-meal tolerance and arginine stimulation tests provide reproducible and complementary measures of β-cell function: results from the foundation for the national institutes of health biomarkers consortium investigative series. Diabetes Care. (2016) 39:1602–13. doi: 10.2337/dc15-0931 PMC5001146 27407117

[13] Institute of Medicine, National Academy of Sciences. Dietary Reference Intakes (2020). Available online at: https://ods.od.nih.gov/HealthInformation/Dietary_Reference_Intakes.aspx (Accessed October 2022).

[14] Feng T Narayanan S. Imputing missing data in large-scale multivariate biomedical wearable recordings using bidirectional recurrent neural networks with temporal activation regularization, in: 41st Annual International Conference of the IEEE Engineering in Medicine and Biology Society (EMBC), Berlin, GERMANY: IEEE, (2019). 10.1109/EMBC.2019.8856966 31946412

[15] Yoon J Jordon J van der Schaar M. Gain: missing data imputation using generative adversarial nets, in: 35th International Conference on Machine Learning (ICML), Stockholm, SWEDEN: PMLR, (2018). 5689–98

[16] Shilo S Godneva A Rachmiel M Korem T Kolobkov D Karady T. Prediction of personal glycemic responses to food for individuals with type 1 diabetes through integration of clinical and microbial data. Diabetes Care. (2022) 45:502–11. doi: 10.2337/dc21-1048 34711639

[17] Zeevi D Korem T Zmora N Israeli D Rothschild D Weinberger A. Personalized nutrition by prediction of glycemic responses. Cell. (2015) 163:1079–94. doi: 10.1016/j.cell.2015.11.001 26590418

[18] Ke G Meng Q Finley T Wang T Chen W Ma W. Lightgbm: a highly efficient gradient boosting decision tree, in: 31st Annual Conference on Neural Information Processing Systems (NIPS), Long Beach, CA, (2017).

[19] Bergstra J Bardenet Rémi Bengio Y Kégl Balázs. Algorithms for hyper-parameter optimization. Adv Neural Inf Process systems. (2011) 24:2546–54. doi: 10.5555/2986459.2986743

[20] Putatunda S Rama K. A modified bayesian optimization based hyper-parameter tuning approach for extreme gradient boosting, in: 15th International Conference on Information Processing (ICINPRO) - Internet of Things, Bengaluru, INDIA: IEEE, (2019) 1–6. doi: 10.1109/ICInPro47689.2019

[21] Lundberg SM Lee S-I. A unified approach to interpreting model predictions, in: 31st Annual Conference on Neural Information Processing Systems (NIPS), Long Beach, CA: Advances in neural information processing systems. (2017).

[22] Lundberg SM Erion G Chen H DeGrave A Prutkin JM Nair B. From local explanations to global understanding with explainable ai for trees. Nat Mach Intelligence. (2020) 2:56–67. doi: 10.1038/s42256-019-0138-9 PMC7326367 32607472

[23] Lundberg SM Erion GG Su-In L. Consistent individualized feature attribution for tree ensembles. arXiv. (2018) 5:25. doi: 10.48550/arXiv.1802.03888

[24] Campbell TW Wilson MP Roder H MaWhinney S Georgantas RW Maguire LK. Predicting prognosis in covid-19 patients using machine learning and readily available clinical data. Int J Med Informatics. (2021) 155:104594. doi: 10.1016/j.ijmedinf.2021.104594 PMC8459591 34601240

[25] Gallwitz B. Implications of postprandial glucose and weight control in people with type 2 diabetes understanding and implementing the international diabetes federation guidelines. Diabetes Care. (2009) 32:S322–S5. doi: 10.2337/dc09-S331 PMC2811482 19875573

[26] Popova P Castorino K Grineva E Kerr D. Gestational diabetes mellitus diagnosis and treatment goals: Measurement and measures. Minerva Endocrinologica. (2016) 41:421–32. doi: 10.1055/a-1284-6011 26824326

[27] Karim RAH Vassanyi I Kosa I. After-meal blood glucose level prediction using an absorption model for neural network training. Comput Biol Med. (2020) 125:103956. doi: 10.1016/j.compbiomed.2020.103956 32861049

[28] Li K Liu C Zhu T Herrero P Georgiou P. Glunet: A deep learning framework for accurate glucose forecasting. IEEE J Biomed Health informatics. (2019) 24:414–23. doi: 10.1109/JBHI.6221020 31369390

[29] Korem T Zeevi D Zmora N Weissbrod O Bar N Lotan-Pompan M. Bread affects clinical parameters and induces gut microbiome-associated personal glycemic responses. Cell Metab. (2017) 25:1243. doi: 10.1016/j.cmet.2017.05.002 28591632

[30] Pustozerov EA Tkachuk AS Vasukova EA Anopova AD Kokina MA Gorelova IV. Machine learning approach for postprandial blood glucose prediction in gestational diabetes mellitus. IEEE Access. (2020) 8:219308–21. doi: 10.1109/Access.6287639

[31] Mendes-Soares H Raveh-Sadka T Azulay S Edens K Ben-Shlomo Y Cohen Y. Assessment of a personalized approach to predicting postprandial glycemic responses to food among individuals without diabetes. JAMA Network Open. (2019) 2:e188102. doi: 10.1001/jamanetworkopen.2018.8102 30735238 PMC6484621

[32] Tily H Patridge E Cai Y Gopu V Gline S Genkin M. Gut microbiome activity contributes to prediction of individual variation in glycemic response in adults. Diabetes Ther. (2022) 13:89–111. doi: 10.1007/s13300-021-01174-z 34799839 PMC8776936

[33] Gonzalez-Rodriguez M Pazos-Couselo M Garcia-Lopez JM Rodriguez-Segade S Rodriguez-Garcia J Tunez-Bastida C. Postprandial glycemic response in a non-diabetic adult population: the effect of nutrients is different between men and women. Nutr Metab. (2019) 16:1–9. doi: 10.1186/s12986-019-0368-1 PMC6637571 31346341

